# Gender Interaction in Association of Perceived Social Support and Health-Related Quality of Life Among Iranian Older People: A Cross-sectional Survey

**DOI:** 10.34172/hpp.2022.08

**Published:** 2022-05-29

**Authors:** Maryam Tajvar, Astrid Fletcher, Emily Grundy, Badrye Karami, Fatemeh Mohabbati

**Affiliations:** ^1^Department of Health Management and Economics, School of Public Health, Tehran University of Medical Sciences, Tehran, Iran; ^2^Faculty of Epidemiology & Population Health, London School of Hygiene and Tropical Medicine, London, United Kingdom; ^3^Institute for Social and Economic Research, University of Essex, Essex, United Kingdom; ^4^School of Public Health, Zabol University of Medical Sciences, Zabol, Iran

**Keywords:** Social support, Quality of life, Aged, Gender identity

## Abstract

**Background:** The purpose of this study was to examine the relationship between perceived social support (PSS) and dimensions of health-related quality of life (HRQoL) and to examine possible gender interaction in the mentioned associations.

**Methods:** A community-based cross-sectional study conducted among 644 participants over the age of 60 years old in Tehran. The data were collected through face-to-face interviews conducted in their own homes, by using a structured multi-sectional questionnaire. The version 1 of the SF-12 scale was used to measure the HRQoL, consisting of two summary measures; PCS (Physical Component Score) and MCS (Mental Component Score). The Persian version of the Social Provisions Scale (SPS) was used to measure PSS. Four multilevel mixed-effects logistic regression models were used to examine the associations.

**Results:** Older people with poor SPS score were 1.8 times more likely to be in the worst quartile of the MCS distribution (CI=1.11-2.93, *P* =0.021), and twice as likely to be in the worst quartile of the PCS distribution (CI=1.18-3.54, *P* =0.011). We found strong evidence to support the hypothesis of gender interaction in the association between economic status and PCS [Men: OR 0.28, CI (0.11-0.71); Women: OR 1.00, CI (0.53-1.88); *P* of Interaction 0.021], and a borderline evidence for gender interaction in the association between physical activity and PCS [Men: OR 5.32, CI (2.14-13.20); Women: OR 1.80, CI (0.82-3.93); *P* of Interaction 0.051].

**Conclusions:** Social support could be regarded as one of the main social determinants affecting HRQoL among older people. Men with poor economic status and poor physical activity, compared to women, are more likely to suffer from poor quality of life, thus men should be prioritized in financial support and life style and physical activity interventions.

## Introduction

Population ageing in the world is unprecedented: by 2050, the number of people of 60 years and older in the world will exceed the number of young people under 15 years for the first time in history.^1,2^ Iran has experienced one of the fastest fertility transitions globally,^3^ so that, by 2030, the elderly population of Iran will increase from 5.6% to 17.5%^4^ and life expectancy at birth will increase to 78.2 years.^5^ Population ageing is usually associated with increased disability and frailty in society.^6^ Thus, provision of health care would be one of the most important issues in the future of Iran and that would be essential to pay attention to this age group, while improving their quality of life (QOL).

The QOL is an increasingly popular concept in the nursing and medicinal sciences.^7^ It was defined by Joyce’s as “how good or bad you feel your life to be”.^8^ QOL is a subjective perception that represents different things to different people.^9^ QOL index is one of the measurable criteria for determining the needs and health conditions of the older people.^10^ Measuring HRQOL is a way of assessing the health status of adults, and it can be used to identify factors that are associated with good health and maintaining performance and improving QOL.^11^ HRQOL, on the other hand, is defined as “how well a person functions in their life and his or her perceived wellbeing in physical, mental, and social domains of health”.^12^ It is considered as one of the important indicators of the outcome of treatment and care interventions in the elderly.^13^ Based on the earlier studies,^14,15^Health-related quality of life (HRQoL) in different populations are associated with various factors including age, gender, education, socio-economic status (SES), outdoor leisure activity, physical activity, social support (SS),^16^ type and duration of residence,^17^ comorbidities,^18^ and being afflicted by chronic diseases.^19^ Among these factors, SS is a key predictor of HRQoL, on which studies have greatly multiplied in last years.^16,20^ Social support, in definition, is an exchange of sources between at least two people which is recognized by the supplier or the recipient to be intended to enhance the well- being of the recipient.^21^ SS in older people is conceptualized as received and perceived support.^22^ “Perceived social support” (PSS) is defined as one’s overall impressions on whether social network is supportive enough or not”.^23^ Due to functional limitations, loss of income sources, and loss of spouse and confidants, SS might be even more essential in old age.^24^ There is evidence that, lack of which are associated with higher mortalities cognition, depressive symptoms, and well-being.^25^ This reliance sensation among elderly may help them with strengthening their coping abilities to overcome the stressors of life.

In addition to age, gender can also influence these concepts, but as various evidences show, different studies do not have the same results on SS and HRQoL in men and women. In some studies, the level of PSS in women is higher than men.^26,27^ But in Karakoç and Yurtsever’s study, men perceived more SS than women.^28^ In Hann’s study, the PSS did not show a significant difference between men and women.^29^ There are limited studies on relationships between HRQoL and gender. The results of the study of Bani Fatemeh et al^30^ indicate that there is a significant difference between in HRQoL of men and women and men’s scores were higher.

Multiple studies conducted to examine factors affecting SS and HRQoL among older adults in other countries^31,32^ and there is little such evidence in Iran.^33^ It is possible that differences in cultural and societal conditions, make it difficult to generalize study findings from those countries to non- western communities like Iran. In addition, none of the previous studies has examined the strength of relationships among PSS and HRQoL in older population, controlling for the effects of potential individual-level and community-level covariates, based on the literature, in separate analysis. The purpose of this study, thus, was to examine the relationship between PSS and HRQoL, with the hypothesis of lower levels of PSS are associated with poorer HRQoL among Iranian older adults. Additionally, interaction of gender and some other factors in the analysis of associations between PSS and HRQoL were examined. Finally, associations between community-level SES with HRQoL were checked. The results of the present study can be used in planning to improve the physical and mental aspects of health, quality and living standards of the older people and improve their social conditions.

## Material and Methods

### Study design and data collection

A cross-sectional community-based survey conducted among 644 participants over the age of 60 years old as a representative sample of elderly living in Tehran. The required sample size was calculated at 800 based on an alpha level of 0.05 and power of 80% using an expected odds ratio (OR) of 2 and a design effect of 1.5 based on results from earlier studies.^34-36^ Study population selected by the sampling method of multistage stratified clustering, in order to guarantee the representation of older people from neighborhoods of different SES. For the first stage of the sampling, three municipal districts of Tehran from areas with different SES were chosen. Then, from each district, one neighborhood was selected, randomly. Probability proportionate to size allocation method was used for randomly selecting people within study cluster. A structured multi-sectional questionnaire was used for data collection through face-to-face interviews conducted at their own homes. The response rate was 76%. There were not any systematic differences between characteristics of no respondents include age and gender compared with respondents.. The results of this study are not generalizable to those older people who are resident in institutions, hospitalized at the time of the survey or those living in other cities than Tehran. Those older people who were unable to participate due to severe mental and physical issues were also excluded for this study.

An identification letter and card for fieldworkers was obtained from Tehran University of Medical Sciences (TUMS) before starting the study. The purpose of the interview described for participants. Fieldworkers gave assurances to participants that the data collected would be treated as confidential and will only be used in anonymously and only for research purposes. Participants’ freedom to discontinue participation during the interview or later was stated in the information sheets. The consent forms had to be signed or fingerprinted before interviews. Anonymity and confidentiality of all participants was ensured through numeric coding during all stages of the research. Questionnaires contained no identification of participants.

### Measurement of Study Variables 

In this study, HRQoL is the outcome variable and the SPS is the main independent variable. A long list of covariates were also measured and their effect on the associations between the main independent and dependent variables were controlled. To achieve the objectives of this research, the structured multi sectional questionnaire was used. It consists of three main parts: 1) individual-level socio-demographic characteristics of participants (including age, gender, number of children, marital status, economic status, employment, education …), in addition to functional health of participants (measured using the Nagi score), which was derived from a series of questions included in the dimension of “Physical functioning”, as a part of the SF-36 scale. The Nagi scale includes 10 types of functions (such as lifting or carrying groceries, climbing one flights of stairs, etc), which might be limited because of physical health conditions. These questions themselves evolved from the pioneering work of Saad Nagi (1965) on the conceptualization of the disability process which was used by World Health Organization (WHO) in the derivation of International Classification of Impairments, Disabilities and Handicaps^37^ and community-level SES, and finally questionnaires to measuring the HRQoL and PSS.

To measure the HRQoL, we used the version 1 of the 12-item Short Form Health Survey (SF-12), as a shorter alternative to the SF-36. The SF-12 is a self-reported multipurpose short form survey.^12^ Cross-cultural validation studies have shown substantial correlations between the summary measures of the SF-36 and the SF-12 Health Survey. The result of SF-12 is reported by two summary measures: the physical component score (PCS) and the mental component score (MCS). A global score is derived from the sum of these two components, estimating an individual’s perception of global HRQoL. Montazeri et al^38^ adopted this scale for Iranian cultural characteristic. The validity and reliability of the SF-12 version1 was checked and suggested that the SF-12 version1 is a reliable and valid measure of the HRQoL in Iranian population. In this study a sample of 5587 people (2721 males and 2866 females) randomly from the general population aged 15 years and older living in Tehran, completed the SF-12 questionnaire. The results showed satisfactory internal consistency for both summary criteria, which are the PCS and the MCS. Cronbach’s α was 0.73 and 0.72 for PCS12 and MCS-12, respectively. Comparison of known groups showed that SF-12 was well differentiated between men and women and individuals who were different in age and educational status (*P* < 0.001). Finally, the analysis of the main components showed a two-factor structure (physical and mental health), which together accounted for 57.8% of the variance. Confirmatory factor analysis also showed that it fits well with the data of two hidden structures (physical and mental health). This questionnaire assesses four of the health concepts (physical functioning, role physical, role emotional and mental health) using two items for each, whereas the remaining four concepts (bodily pain, general health, vitality and social functioning) are represented by a single item. All 12 items are used to calculate the PCS-12 and the MCS-12.^39^

To select a suitable instrument for measuring the PSS, a variety of available scales was reviewed^40-42^ and finally, the Persian version of the Social Provisions Scale (SPS) originally developed by a research group at University of California at Los Angeles^43^ based on the Weiss’s (1974) model of social provision, was selected. PSS consist of six dimensions including: (1) Provision for attachment, provided most often by marriage or other cross-sex relationships; (2) Social integration, provided by a network of friends and colleagues who offer companionship and opportunity to share interests and values; (3) Opportunity for nurturing behavior, provided most often to children which develops a sense of being needed; (4) Reassurance of worth; (5) Guidance, provided most often by supportive friends and relatives; (6) Reliable alliance, provided most often by kin relationships. This scale has psychometric properties, contains simply worded questions and is relatively brief.^43^ Also it has been recommended as a suitable scale for older people.^39^ This scale has been translated to Persian by Zaki^44^ in Iran as a reliable and valid instrument. Based on this study, the SPS has highly significant validity and reliability and is suitable for Iranian culture, although this scale was not validated in older people in Iran. The SPS consisted of 24 items with a four-point Likert scale, ranging from “strongly disagree” to “strongly agree”. Each item is placed in one of six dimensions as listed above. A total score for each dimension is computed, as the mean of the scores of the items falls in the range 4-16. Overall, the range of scores of the SPS was 24-96, with higher scores indicating a more PSS.

### Statistical analysis

After gathering data from the 644 participants who completed the questionnaires, the data were entered into STATA (Release 14. College Station, TX: StataCorp LP) for analyses. Analyses started with descriptive statistics to summarize the data. For continuous variables with normal distribution, mean and standard deviation, or where relevant median and inter quartile range were used for presentation of the data. Categorical variables were presented using numbers and percentages of groups. The results of the descriptive analysis are presented in descriptive tables. Then univariable and multivariable logistic regression analyses were used to analyze associations between indicators of the HRQoL and the PSS. The data of this study were clustered and had a hierarchical structure, so that individuals in the study were nested within households within neighborhoods within districts of Tehran. Therefore, multilevel models, including mixed-effects logistic regression model, instead of simple models, were selected, as the outcome measure was binary variable. was used.

In order to test the hypothesis of this research, the mixed-effect model was arrived at through a number of modeling stages. In all models, only questionnaires with complete data on all variables were included. In the first model, the association of the PSS (main independent variable) and other covariates with the HRQoL (dependent variable) were initially assessed one by one in a mixed-effect model adjusted for age and gender. The selection of covariates was based on the conceptual approach informed by theoretical considerations and results of the literature review. Then correlation among independent variables were checked using a correlation matrix and one of the highly correlated variables in any pair was dropped from further multivariable analyses. In the second model, other covariates were added to the first model to check how the association between main independent and dependent variables changes in the presence of other covariates. The third model was the same as Model 2 but with an additional section to check the possible gender interaction. In addition, in the final model, community-level data were added to a model with the main exposure, age and gender and then to the model of stage 2 in order to explore how associations between independent and dependent variables vary according to community-level characteristics.

## Results

Men and women comprised exactly the same number in this study (322 men and 322 women). Descriptive profile of the study participants for sociodemographic variables are available from our previous paper published from the same data.^16^ The characteristics of study sample were similar to the same data in national census data for similar age group in the city of Tehran.

Table 1 shows the descriptive statistics on the HRQoL. After scoring of the SF-12, the mean (SD) of the PCS and the MCS were calculated at 43 (29) and 53 (22) respectively indicating considerably better mental health than physical health among participants. The HRQoL of men was better than that of women in both components; the PCS (mean 52 vs. 34) and the MCS (mean 59 vs. 47). As Table 1 shows, women were twice more likely to be in the worst quartile of both the PCS and the MCS. Age was also associated with HRQoL; being older was more likely to be in the worst quartile of the HRQoL, especially in the PCS scores.

**Table 1:** Distribution of participants in the worst and other quartiles of the PCS and MCS (SF-12), by gender and age group

No. (%) *	**PCS**		**MCS**	
	**Worst quartile**	**Rest quartiles**	**Worst quartile**	**Rest quartiles**
Gender,				
Men (n = 322)	53 (17.1)	256 (82.8)	56 (17.5)	263 (82.5)
Women (n = 322)	104 (32.9)	212 (67.1)	111 (35.1)	205 (64.9)
Age groups				
60-69 (n = 329)	48 (15.0)	272 (85.0)	64 (19.6)	263 (80.4)
70-79 (n = 244)	71 (30.2)	164 (69.8)	80 (33.6)	158 (66.4)
80 + (n = 71)	38 (55.9)	30 (44.1)	23 (33.8)	45 (66.2)

* The sum of numbers in some rows is less than total people in that row because of item non-response.

The mean (SD) score for the PSS, as measured by the SPS, was calculated at 71.8 (9.7) (men 72.5, women 71.2) with a range of 24–96. A higher proportion of women than men (58% versus 42%) had scores in the worst quartile of the SPS. The highest score for both men and women was in the dimension of ‘attachment’ and the lowest score was in the dimension of ‘social integration’ (Table 2).

**Table 2:** Mean and SD of scores of the SPS and its dimensions to measure PSS of participants, by gender

**Mean (SD)**	**Total SPS** **(Score range 24-96)**	**Reliable alliance**	**Attachment**	**Guidance**	**Opportunity for nurturance**	**Social Integration**	**Reassurance of worth**
Men	72.5 (9.0)	12.4 (2.1)	12.9 (1.9)	11.8 (2.0)	12.5 (2.3)	11.0 (1.9)	11.6 (1.7)
Women	71.2 (10.3)	12.6 (2.5)	12.8 (2.4)	11.2 (2.6)	11.7 (2.6)	10.7 (2.3)	11.7 (1.8)
Total	71.8 (9.7)	12.5 (2.3)	12.8 (2.2)	11.5 (2.4)	12.1 (2.4)	10.9 (2.1)	11.7 (1.8)

The results of the association between SPS and two subscales of HRQoL (measured by SF-12) including the MCS and the PCS, which cannot be combined, are shown in two separate Tables as 3 and 4.In this analysis, the SPS scores were dichotomized into the lowest score quartile (27%) versus the rest, as we were interested to check whether low SPS scores were associated with poorer HRQoL.

**Table 3:** Mixed-effects logistic regression models for analysing the association of PSS and MCS (worst quartile of the MCS distribution)

**Variables**	Model 1**		**Model 2**		**Model 3**		**Model 4**	
	**Univariable** **each factor+age+gender**		**Multivariable** **SPS+other individual-level covariates**		**Model 2+interaction** **of gender**		**Model 2+community-level covariate**	
	**OR (95% CI)**	P*	**OR (95% CI)**	***P***	**OR (95% CI)**	***P***	**OR (95% CI)**	***P***
Individual-level variables								
Perceived social support (PSS)								
Rest quartiles	Ref.		Ref.		Ref.		Ref.	
Worst quartile	2.66 (1.57-4.50)	**<0.001**	1.80 (1.11-2.93)	**0.021**	1.76 (1.09-2.85)	**0.021**	1.84 (1.11-3.04)	**0.021**
Age								
Continuous	1.05 (1.02-1.09)	**0.002**	0.98 (0.95-1.02)	0.401	0.98 (0.95-1.02)	0.401	0.98 (0.95-1.02)	0.391
Gender								
Man	Ref.		Ref.		-		Ref.	
Woman	3.08 (1.85-5.14)	**<0.001**	1.84 (1.12-3.03)	**0.011**	-	-	1.82 (1.10-3.01)	**0.021**
Ethnicity								
Fars (main ethnicity)	Ref.		-		-		-	
Non-Fars (minor ethnicities)	1.02 (0.68-1.53)	0.931	-	-	-	-	-	-
Religious beliefs								
Strong	Ref.		-		-		-	
Less strong	1.50 (0.74-3.02)	0.261	-	-	-	-	-	-
Married or not								
No	Ref.		-		-		-	
Yes	0.72 (0.4-1.18)	0.191	-	-	-	-	-	-
Having children								
No	Ref.		-		-		-	
Yes	0.74 (0.23-2.34)	0.611	-	-	-	-	-	-
Family size								
0-10 members	Ref.		-		-		-	
11 + members	0.88 (0.49-1.59)	0.681	-	-	-	-	-	-
Living arrangement								
Living alone/others	Ref.		-		-		-	
Living with child/children only	0.61 (0.30-1.24)	0.371	-		-		-	-
Living with spouse only	0.65 (0.33-1.26)		-	-	-	-	-	-
Living with spouse& child/children	0.56 (0.28-1.10)		-		-		-	
Quality of relationships with spouse***								
Less than very good	Ref.		-		-		-	
Very good	0.65 (0.41-1.04)	0.071	-	-	-	-	-	-
Quality of relationships with at least 1 family member								
Less than very good	Ref.		-		-		-	
Very good	0.62 (0.37-1.05)	0.081	-	-	-	-	-	-
Education								
Illiterate	Ref.		-		-		-	-
1-9 years	1.00 (0.63-1.57)	0.991	-	-	-	-	-	-
10 years and more	0.98 (0.50-1.95)		-		-		-	
Economic status perceived								
Poorer than average	Ref.		Ref.		Ref.		Ref.	
Same or better than average	0.58 (0.36-0.94)	**0.021**	0.79 (0.50-1.25)	0.331	0.81 (0.51-1.28)	0.371	0.83 (0.51-1.30)	0.381
Having medical insurance								
Yes	Ref.		-		-		-	
No	1.58 (0.84-3.00)	0.161	-	-	-	-	-	-
Participating in social activities								
0-2 activities out of 10 (poor)	Ref.		Ref.		Ref.		Ref.	
3-10 activities (non poor)	0.13 (0.05-0.36)	**<0.001**	0.29 (0.12-0.68)	**0.005**	0.30 (0.13-0.69)	**0.005**	0.29 (0.12-0.70)	**0.006**
Smoking								
Regularly/Sometimes	Ref.		-		-		-	
Never	1.03 (0.58-1.81)	0.921	-	-	-	-	-	-
Physical activity (exercise)								
Regularly/Sometimes	Ref.		Ref.		Ref.		Ref.	
Never	2.03 (1.23-3.34)	**0.005**	1.27 (0.77-2.10)	0.341	1.23 (0.75-2.04)	0.411	1.35 (0.80-2.29)	0.251
Disability limiting daily life								
Yes	Ref.		-		-		-	
No	0.22 (0.11-0.45)	**<0.001**	-	-	-	-	-	-
Nagi (functional health)								
Rest quartiles	Ref.		Ref.		-		Ref.	
Worst quartile	5.15 (2.47-10.74)	**<0.001**	3.66 (1.89-7.12)	**<0.001**	-	-	3.87 (1.94-7.72)	**<0.001**
Interaction of Nagi and Gender P						0.301		
Nagi in men								
Rest quartiles	-		-		Ref.		-	
Worst quartile	-	-	-	-	4.89 (1.97-12.10)	**0.001**	-	-
Nagi in women								
Rest quartiles	-		-		Ref.		-	
Worst quartile	-	-	-	-	3.07 (1.52-6.18)	**0.002**	-	-
Community-level variable								
SES of neighbourhood								
Poor	Ref.		-		-		Ref.	
Middle	1.17 (0.73-1.87)	0.801	-	-	-	-	1.18 (0.70-1.98)	0.401
High	1.06 (0.61-1.83)		-		-		1.58 (0.81-3.07)	
LR test vs. logistic regression *P*-value		0.99	0.99	0.96
Neighbourhood-level ICC		≈ 0	≈ 0	≈ 0
Household-level ICC		0.09	0.06	0.14

LR, likelihood-ratio te st; ICC, intra-class correlation *P value of OR reported for each dummy variable compared to the baseline category controlled for other variables. For categorical variables overall *P* value was reported using ‘testparm’ in STATA. In all models only people with complete data on all variables were included. **In Model 1, ICC for levels of analysis were not reported as separate univariable models were fitted for each variable and each model had different ICC for each level. ***For these variables, analysis excluded those without a spouse/ a child.

**Table 4:** Mixed-effects logistic regression models for analysing the association of PSS and PCS (worst quartile of the PCS distribution)

**Variables**	Model 1**		**Model 2**		**Model 3**		**Model 4**		**Model 5**	
	**Univariable** **Each Factor+Age+Gender**		**Multivariable** **SPS+Other Individual-level Covariates**		**Model 2+Interaction of Gender& Physical Activity**		**Model 2+Interaction of Gender& Economic Status**		**Model 2+Community-Level Covariate**	
	**OR (95% CI)**	***P*** *	**OR (95% CI)**	***P***	**OR (95% CI)**	***P***	**OR (95% CI)**	***P***	**OR (95% CI)**	***P***
Individual-level variables										
Perceived social support (PSS)										
Rest quartiles	Ref.		Ref.		Ref.		Ref.		Ref.	
Worst quartile	2.53 (1.38-4.60)	**0.002**	2.04 (1.18-3.54)	**0.011**	1.92 (1.12-3.27)	**0.021**	1.99 (1.13-3.49)	**0.021**	2.09 (1.19-3.65)	**0.011**
Age										
Continuous	1.14 (1.07-1.21)	**<0.001**	1.08 (1.04-1.13)	**<0.001**	1.08 (1.03-1.12)	**<0.001**	1.08 (1.03-1.13)	**<0.001**	1.08 (1.03-1.13)	**<0.001**
Gender										
Man	Ref.		Ref.		-		-		Ref.	
Woman	4.33 (1.07-9.20)	**<0.001**	2.36 (1.29-4.33)	**0.005**	-	-	-	-	2.18 (1.20-3.94)	**0.011**
Ethnicity										
Fars (main ethnicity)	Ref.		-		-		-		-	
Non-Fars (minor ethnicities)	1.13 (0.66-1.93)	0.641	-	-	-	-	-	-	-	-
Married or not										
No	Ref.		-		-		-		-	
Yes	0.82 (0.44-1.53)	0.541	-	-	-	-	-	-	-	-
Having children										
No	Ref.		-		-		-		-	
Yes	0.87 (0.19-3.96)	0.861	-	-	-	-	-	-	-	-
Living arrangement										
Living alone/others	Ref.		-		-		-		-	
Living with children only	0.87 (0.37-2.06)	0.841	-	-	-	-	-	-	-	-
Living with spouse only	0.91 (0.40-2.07)		-		-		-		-	
Living with spouse& children	0.70 (0.30-1.65)		-		-		-		-	
Education										
Illiterate	Ref.		Ref.		Ref.		Ref.		Ref.	
1-9 years	0.74 (0.41-1.35)	0.121	1.15 (0.67-1.98)	0.561	1.12 (0.65-1.90)	0.521	1.11 (0.63-1.95)	0.771	1.03 (0.58-1.84)	0.211
10 years and more	0.33 (0.11-0.95)		0.70 (0.26-1.85)		0.65 (0.25-1.70)		0.79 (0.29-2.14)		0.38 (0.11-1.26)	
Economic status perceived										
Poorer than average	Ref.		Ref.		Ref.				Ref.	
Same or better than average	0.44 (0.24-0.80)	0.008	0.63 (0.37-1.05)	0.081	0.63(0.38-1.05)	0.081			0.59(0.34-1.02)	0.061
Having medical insurance										
Yes	Ref.		-		-		-		-	
No	1.14 (0.50-2.61)	0.751	-	-	-	-	-	-	-	-
Participating in social activities										
0-2/10 activities (poor)	Ref.		-		-		-		-	
3-10/10 activities (non poor)	0.09 (0.02-0.35)	**0.001**	-	-	-	-	-	-	-	-
Smoking										
Regularly/Sometimes	Ref.		-		-		-		-	
Never	0.93 (0.45-1.92)	0.841	-	-	-	-	-	-	-	-
Physical activity(exercise)										
Regularly/Sometimes	Ref.		Ref.		-		Ref.		Ref.	
Never	4.09 (1.94-8.63)	**<0.001**	3.18 (1.63-6.20)	**0.001**	-	-	3.27 (1.62-6.60)	**0.001**	3.33 (1.68-6.60)	**0.001**
Interaction of Physical activity & gender *P*				**0.05**					
Physical activity in men									
Regularly/Sometimes		-		Ref.		-		-	
Never		-	-	5.32 (2.14-13.20)	**<0.001**	-	-	-	-
Physical activity in women									
Regularly/Sometimes		-		Ref.		-		-	
Never		-	-	1.80 (0.82-3.93)	0.141	-	-	-	-
Interaction of Economic status & Gender *P*						**0.021**			
Economic status in men									
Poorer than average		-		-		Ref.		-	
Same or better than average		-	-	-	-	0.28 (0.11-0.71)	**0.007**	-	-
Economic status in women									
Poorer than average		-		-		Ref.		-	
Same or better than average		-	-	-	-	1.00 (0.53-1.88)	0.991	-	-
Community-level variable										
SES of neighbourhood										
Poor	Ref.		-		-		**-**		Ref.	
Middle	0.72(0.39-1.34)	0.541	-	-	-		**-**		0.86 (0.48-1.53)	0.161
High	0.78(0.38-1.58)		-	-	-	-	**-**	**-**	2.11 (0.86-5.18)	
LR test vs. logistic regression *P* value		0.69	0.79	0.62	0.65
Neighbourhood-level ICC		≈0	≈0	≈0	≈0
Household-level ICC		0.22	0.20	0.24	0.22

LR, likelihood-ratio te st; ICC, intra-class correlation *P value of OR reported for each dummy variable compared to the baseline category controlled for other variables. For categorical variables, overall *P* value was reported using ‘testparm’ in STATA. In all models, only people with complete data on all variables were included. **In Model 1, ICC for levels of analysis were not reported as separate univariable models were fitted for each variable and each model had different ICC for each level

The analysis of association between SPS and MCS (Table 3) in the first model showed that those in the worst quartile of SPS were 2.7 times more likely to be in the worst quartile of the MCS (*P* < 0.001). Age, gender, economic status perceived, social participation, physical activity, disability and Nagi score showed also significant associations with the MCS. After dropping the highly correlated covariates and those not showing significant contribution based on the results of Model 1 and likelihood-ratio (LR) tests, Model 2 was run with the refined variables. In this model, although the OR of SPS in association with MCS was nearly halved compared to the earlier model, the association was still significant (*P* = 0.021). Age lost its association with MCS (*P* = 0.401) and gender’s association was highly attenuated in this model but remained significant (*P* = 0.011). Social participation and Nagi score were two other factors that remained significant in this model. In particular, Nagi score showed the most important contribution in association with MCS, suggesting that being socially active was a protective factor (OR = 0.29, *P* = 0.005) but having poor physical health was a risk factor for MCS (OR = 3.7, *P* < 0.001). In gender-specific analysis, the factor with the greatest difference between men and women was the Nagi score, which was most significantly associated with men’s MCS than women. Thus, we formally tested for the interaction of gender in the association between Nagi and MCS in Model 3. There was no significant interaction between gender and Nagi score however (*P* = 0.301). Association of other variables in Model 3 were almost the same as for Model 2. In the final model, the association between SPS and MCS was slightly stronger than the two previous models but the p-value was the same as before (OR = 1.84, *P* = 0.021). In this model, being female had a slightly smaller association with MCS (OR = 1.82, *P* = 0.021). Poor Nagi score and higher social participation had the same effects as before. As with the previous models, the association between SPS and other aspects of health in this objective showed no significant association between SES of neighborhood and MCS.

Analysis for association between SPS and PCS are shown in Table 4. In Model 1, those in the worst quartile of SPS were 2.5 times more likely to be in the worst quartile of PCS (*P* = 0.002). Age, gender, economic status perceived, social participation, and physical activity also showed significant associations with PCS, similar to factors associated with MCS as explained previously. In Model 2, when other covariates were included, the OR of being in the worst quartile of the SPS in association with the worst quartile of the PCS was decreased to 2 fold, but still significant (*P* = 0.011). In this model, of other covariates, age, gender and physical activity were also factors showing significant associations with PCS. As shown in Model 3 and Model 4 of Table 4, there was strong evidence (*P* = 0.021) to support the hypothesis of gender interaction in the association between economic status and PCS, and borderline evidence (*P* = 0.051) for the hypothesis that gender modified the association between physical activity and PCS. These analyses showed that the men who rarely took part in physical activity had a much greater chance of reporting worst PCS compared to women with the same level of physical activity (OR 5.3 vs. 1.8). Additionally, having a better economic status was found to be important for men’s PCS, while women with poor or non-poor economic status reported similar PCS scores (OR 0.3 vs. 1.0). In the last model, when the effect of SES of neighbourhood was included, there was even stronger evidence compared to interaction models to support the significant association between SPS and PCS (OR = 2.09, *P* = 0.011). The association of other factors in Model 5 with PCS was similar to before, but individual SES (economic status) here showed more association with PCS with borderline evidence (OR = 0.6, *P* = 0.061), while neighbourhood SES had no significant effect. The analysis showed that a high quality relationship with family members are associated with PSS of older people. Moreover, PSS showed significant associations with HRQoL.

## Discussion

In this study, we examined the influence of the PSS on HRQoL (using the SF-12) among older people. Our results showed that those placed at the worst quartile of the SPS score, versus other quartiles, were 1.8 times more likely to be in the worst quartile of the MCS distribution (*P* = 0.021), and twice as likely to be in the worst quartile of the PCS distribution (*P* = 0.011) (Tables 3 and 4). These findings corroborated other studies conducted in Iran indicating a positive association between PSS and HRQoL,^20^ and emotional SS and HRQoL.^45^ Also the findings of our study are consistent with prior research on the association of the PSS and HRQoL among Turkish^46,47^ and Israeli adults and older people.^48^ In the study of Filazoglu and Griva^46^ PSS showed the strongest association with MCS and the second strongest association with the PCS among all other variables in regression models. The results of a longitudinal study in Israel^49^ found SS as to have a stable, significant contribution to the QoL of participants in all three-time points of this study.

Similar toour study, Aghayari Hir and colleagues’^50^ study showed a significant difference in HRQoL by gender, meaning that men’s HRQoL score was higher than women. This finding is in line with the results of the studies of Hui-Chuan, who concluded in their study that older women achieved lower scores on HRQoL in all its dimensions.^51^ Given the impact of SS from friends on the QOL of the elderly, it can be inferred that this type of support creates a network of belonging and connection for the elderly that makes the elderly feel respected and self-worth and because they can express their emotions to their friends, they feel healthy and alive.^50^ This issue can be explained by the cultural and social factors in society. It can be argued that women may have shortcomings and pressures that affect their overall QOL due to cultural issues and social constraints in various areas of QOL such as physical, psychological and social. ^52^

Few studies have considered the mechanisms whereby PSS may act positively to improve HRQoL. It is suggested that PSS may help people with strengthening their coping abilities to overcome the stressors of life or may have direct effect on their HRQoL through biological, psychological or behavioural pathways. Helgeson believes that SS improves and enhances the QOL and mentioned that SS not only improves mood, but also encourages people to participate in social activities.^53^ Regarding people with different illnesses, Filazoglu and Griva^46^ discussed that SS can directly influence their adjustment process and HRQoL in two ways; (*i*) tangible or instrumental SS which may aid patients in the process of physical recovery or in dealing with the physical challenges of illness and associated treatments hence allowing them to maintain a reasonable level of physical aspect of HRQoL (PCS); (*ii*) emotional support which can provide reassurance in the process of dealing the emotional disorder of illness diagnosis and treatment and by making patients feel loved and valued in these times of hardship hence minimising emotional distress and impairments in mental aspect of HRQoL (MCS). According to some studies,^47,54^ low levels of SS tend to be associated with poorer PCS and MCS of chronically ill diabetic patients, while higher levels of SS contribute to better adjustment, a sense of purpose, less uncertainty and better compliance and control of these patients.

Further, the analysis in this study showed that the strength of the association between SPS and MCS decreased considerably in the presence of poor physical functioning status (Table 3), as poor physical functioning showed the strongest association with poor MCS even after controlling for effects of many other factors in the model (OR = 3.87, *P* < 0.001). Given the reported inverse relationship between functional health and QoL^55^ it is not surprising that the SS was less influential on HRQoL when physical functioning is poor. This finding lends support to the importance of managing and improving functional health of older people, as any SS interventions to improve HRQoL of older people would not be effective enough while these people are suffering from poor functioning. However, based on the findings, PSS considerably decreased the strength of harmful effects of poor functional status on HRQoL (MSC). This finding may lend some support to the stress-buffering model of SS as it enables older people to cope better with their poor physical situation, although it was not strong enough to eliminate most negative effects of poor physical health.

In addition, we found that there is a significant interaction of gender in the association between physical activity and the PCS; men with the lowest level of physical activity versus those with regular physical activity were more likely than women in the same situation to be in the worst quartile of the PCS score. This is consistent with the findings of Asfar et al^56^ in Syria indicating that low physical activity is important for men’s health only. This finding may reflect the fact that in conservative societies, physical activity is more feasible for men than women, particularly in old age, where certain recreational activities may not be available for many women. Additionally, in this study we observed that gender can also modify the association between economic status and PCS (*P* = 0.02), so that for men but not women, having a better economic status was associated with a lower risk of being in the worst quartile of PCS. One possible explanation for this finding might be that women’s economic well-being depends more on family or spouse than individual economic position. Another possibility is that the male breadwinner role means that men feel inadequate in some way if their economic status is poor and this affects their health. It is also possible that men with better economic status compared to poorer men had better access to health services or better life style. This is anyway a very complicated subject associating with many other factors.

The main limitation of this study is its cross-sectional design, as in this study design, the temporal associations between SS and health cannot be ascertained and reverse association cannot be excluded. For example, people with poor MCS and PCS may underestimate their SS resources. The temporality of this association could not be determined in this research and longitudinal studies are required. Another limitation is that this study was underpowered to calculate interaction effects. Future research should use larger sample size to allow for analysis of gender interaction and the role of various sources of support in health and QoL of older people. Additionally, the results of this study are generalizable only to community- living older people, but not to institutionalized elderly, and those hospitalized at the time of the survey or older people living in other parts of Iran. Future studies should include the excluded groups for whom associations may differ and have a wider geographical scope. Importantly, further research also needs to elucidate the possible pathways and mechanisms whereby PSS may influence the mental and physical components of HRQoL of elderly in Iran.

## Conclusion

According to findings, strong evidence was found for gender interaction in the association between economic status and physical activity with poor HRQoL; men with poor economic status and poor physical activity, compared to women, are more likely to have poor HRQoL. This emphasizes that older men should be prioritized in financial support and physical activity interventions in future health policy planning and interventions.

Retirement from work, inability to earn money, expenses due to disability and old age, etc. cause the elderly to be financially vulnerable, while these people were the ones who provided for the family expenses before old age and suddenly found themselves in a situation where they are much weaker than before, more affected and feel more powerless. Such issues reduce the happiness of these people and consequently they will experience a lower HRQoL. In addition, due to changing physical needs and reducing the ability and physical function of the elderly, it is necessary to provide facilities in the community in accordance with the needs of the age and create the necessary motivation in these people through training and implementation of motivational programs. Therefore, officials and policy makers at the macro level should pay attention to meeting the needs of the elderly and provide the necessary conditions in this regard so that these people can experience happy, and successful ageing.

## Acknowledgements

We thank Tehran University of Medical Sciences for its partial fund for this research. Also thanks to the participants of this study.

## Authors’ contributions

MT designed the study, gathered data, carried out the statistical analysis and drafted the manuscript under supervision of EG and AF. EG and AF also contributed to interpretation of data significantly. BK and FM helped in literature review and using them in writing up the manuscript. They both also helped in data collection and data entry process.

## Funding

Fund for conducting this study is from Tehran University of Medical Sciences (TUMS) [grant number 8904-27-02-88].

## Ethical approval

The ethical committee of the LSHTM and also the TUMS approved the study protocol (Code 8904-27-02-88). All methods were carried out in accordance with relevant guidelines and regulations. Written informed consent was obtained from all participants prior to the investigation.

## Competing interests

The authors reported no potential conflict of interest.
